# Radiation and mechanical performance of cementitious materials containing ecofriendly nano laboratory waste glass

**DOI:** 10.1038/s41598-024-71468-2

**Published:** 2024-09-19

**Authors:** Mona Elsalamawy, Mona M. Gouda, Israa G. Abdalmawla, Mahmoud I. Abbas, Ahmed M. El-Khatib

**Affiliations:** 1 Structural Engineering Department, Faculty of Engineering, Alexandria University, Alexandria, 21544 Egypt; 2 Physics Department, Faculty of Science, Alexandria University, Alexandria, 21511 Egypt

**Keywords:** Waste glass, Mortar, Scanning electron microscope, Mechanical properties, Linear attenuation coefficient, Nanoscience and technology, Applied physics

## Abstract

This study helps in managing waste glass and greening the environment by incorporating laboratory waste glass into mortar production to make an eco-friendly shielding material against gamma rays. The efficiency of using waste glass powder as a cement replacement or addition in mortar production was studied by using two waste glass sizes: micro glass (particle size range from 10.09 to 24.73 μm) and nano glass (particle size range from 10.57 to 26.42 nm) to design different mortar specimens with varying percentages of fine glass powder from 0 to 30%. Compressive strength and flexure strength were evaluated to determine mechanical properties. The results indicated that adding WGP to mortar positively affects the characteristics of cementitious composites. The linear and mass attenuation coefficients of the samples were experimentally determined using a NaI detector and various radioactive sources (Am-241, Ba-133, Eu-152, Cs-137, and Co-60) with gamma energies ranging from 59.53 to 1332 keV. The obtained coefficients were then compared to the theoretical values of the composites using XCOM software to verify their accuracy. Additionally, the half-value layer, tenth-value layer, mean free path, and effective atomic number were computed. Furthermore, the results revealed that the mortar sample with 30% nano additive glass was the most effective in reducing gamma radiation.

## Introduction

The applications of radiation worldwide in vast fields (industry, engineering, agriculture, medicine and generating electricity) are flourishing. Despite these benefits, ionizing radiation can be dangerous to humans. Due to its ability to remove electrons from atoms, long-term human exposure to ionizing radiation can cause permanent gene mutations, cancer, or death. Therefore, researchers are constantly searching to discover the ideal shielding material for each application to attenuate radiation energy in order to limit the hazards that face humans who are routinely exposed to radiation. Besides following other common safety precautions, such as reducing radiation exposure time and maximizing distance from the source as much as possible^1–5^. Therefore, the development of an efficient radiation shield necessitates the presence of various characteristics in the chosen material, such as density, thickness, thermal properties, and non-toxicity to both humans and the environment. Additionally, it should exhibit resistance to damage and corrosion, while being economically viable^6^. Lead is considered the finest material for absorbing radiation due to its heavy density and high atomic number, but unfortunately, it is not desired due to its poor mechanical strength and its high toxicity^7,8^.

Mortar is widely recognized as one of the most commonly employed materials for shielding against nuclear radiation. This is due to its exceptional ability to attenuate gamma radiation, making it an effective radiation barrier^9^. Moreover, mortar possesses several advantageous qualities, including affordability, durability, eco-friendliness, and widespread availability. Essentially, it is composed of simple ingredients such as cement, sand, and water. Thus, it has been used for many radiation purposes, such as nuclear reactors and facilities for radiotherapy^10^. However, it has drawbacks like cracking and inadequate chemical resistance^11^, hence, it demands further improvement. In order to evaluate the advantages and disadvantages, it is necessary to consider the materials with a higher density than sand that are utilized to enhance the shielding properties of mortar. The density of these materials plays a crucial role in improving the effectiveness of their shielding properties. Seifan et al.^12^ proved that the addition of nano silica in mortar enhanced fresh properties and mechanical strength compared to traditional mortar. Gallala et al.^13^ tested the mechanical and shielding qualities of cement mortar using barite-fluorspar mine waste (BFMW) as a filler. According to their findings, utilizing BFMW aggregates enhances the attenuation coefficient of mortar by 20%. Ghazanlou et al.^14^ proved that magnetite nanoparticles impeded the spread of cracks, and it turns out that using Fe_3_O_4_ nanoparticles with 0.25 wt%, in comparison to plain cement, the porosity dropped by 20% while the compressive, flexural, and tensile strengths increased by 32, 25, and 20%, respectively.

In recent years, a lot of research has focused on applying nanotechnology to radiation shielding. This has led to remarkable developments in getting an improved cementitious shield over the traditional one. A study undertaken by Sayyed et al.^15^ showed that increasing the ratio of Fe_2_O_3_ nanoparticles in mortar leads to a notable improvement in the shielding properties and proved that 0.25 wt% of nano Fe_2_O_3_ has the highest linear attenuation coefficient value. Alresheedi et al.^16^ found that the incorporation of nano bismuth oxide (Bi_2_O_3_) and tungsten trioxide (WO_3_) nanoparticles into mortar enhanced the thermal stability, mechanical strength, and shielding ability of the composite. where the linear attenuation coefficient values rose by up to 2.47 times at 0.06 MeV and 1.12 times at 0.662 MeV compared with the conventional mortar. Also, Sikora et al.^17^ explained that the reduction of Fe_3_O_4_ particles to nano-size can greatly improve in mechanical strength and micro structure of cementitious paste. This is due to the fact that nano particles offer a more uniform distribution throughout the matrix, reducing the gaps in the structure, increasing photon dispersion, and thus leading to higher attenuation^18–20^.

The global community’s interest in recycling has surged in recent years. This is due to the fact that recycling not only addresses the issues of overflowing landfills and environmental pollution, but also plays a crucial role in conserving our precious natural resources. Numerous studies have been conducted to further explore the benefits of recycling^21–24^. Significantly, there are vast quantities of medical waste glassware (beakers, tubes, pipettes, and flasks) that hospitals, pharmaceutical factories, and laboratories of practical colleges produce, which leads to increased environmental pollution. Also, as it has been proven before, glass has superior thermal stability, high resistance to chemical reactions, good transparency for light, easily formable, low costing, has high radiation attenuation, and is available in all areas around the world^25–29^. Patel et al.^30^ and Mehta et al.^31^ proved that fine waste glass powder can emulate the action of sand and therefore can be used as a substitute for sand in mortar. The efficiency of using waste glass powder in cement-based materials depends on many factors, including purity from recycled sources, chemical composition, and particle size distribution^32^. As revealed by recent research conducted by Xiao et al.^33–35^, the vital factor that affects the performance and reactivity of waste glass powder (WGP) is particle size; when the particle size is less than 63 μm, the pozzolanic activity is enhanced. Ahmad et al.^36^ added that the size of glass grains should be fewer than 75 microns, as this size would be sufficient to achieve a good index of pozzolanic activity in waste glass and also improve the internal compactness of mortar by filling holes in the matrix.

This study focused on the reuse of medical waste glass as a filler in cement mortar to improve the mortar's radiation shielding capabilities and mechanical strength. One common application of glass compounds combined with cement in radiation shielding is in the construction of nuclear power plants. These materials are crucial for creating barriers that shield workers and the surrounding environment from the harmful radiation emitted during nuclear reactions. The glass compounds offer durability and strength, while the cement serves to seal any cracks or gaps in the structure, ensuring maximum protection against radiation leaks. When combined, these materials form a robust barrier that effectively blocks and absorbs harmful rays, making it safe for workers to operate within the plant without risking exposure to dangerous levels of radiation. This innovative application of glass compounds with cement showcases how advanced materials can be utilized to enhance safety measures in high-risk environments like nuclear power plants. The research was conducted in three main sections. Firstly, various mortar mixes were tested to determine the optimal percentage and particle size of waste glass powder (WGP) by evaluating compressive strength and flexural strength. Secondly, the study explored how reducing the size of glass particles from micro to nano affects the attenuation of gamma rays in the energy range of 0.05951–1.332 MeV. The radiation performance of the composite was compared based on the mixing method, whether through replacement or addition. Lastly, the results of lead attenuation were used as a reference point to analyze the findings of the study.

## Materials

### Cement

**Fig. 1:**
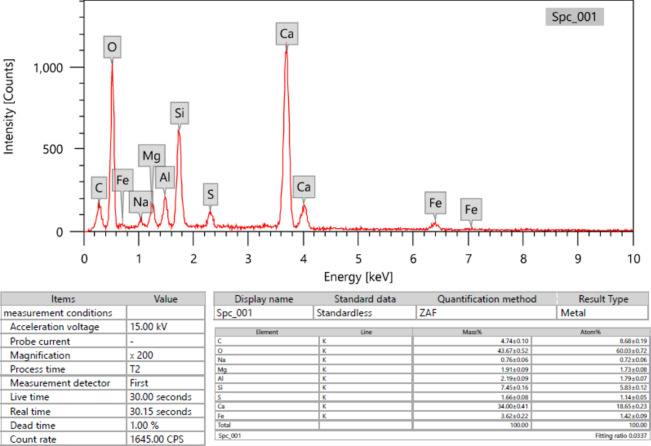
Energy dispersive X-ray analysis (EDX) of cement.

Portland cement (CEM II/A-P 42.5N) was used in this research. The physical properties of cement, setting time, and soundness were determined according to EN 196-3:2005@@^37^, while the determination of compressive strength was conducted according to EN 196-1:2005@@^38^. The chemical composition of cement was determined using XRF analysis. The physical characteristics and chemical composition of cement are presented in Table 1. Also, energy dispersive X-ray analysis (EDX) of cement is shown in Fig. 1.

**Table 1:** Physical and chemical composition of cement “CEM II/A-P 42.5N”.

	Physical properties		EN 197-1/2013^22^ Specification limits
Surface area (cm^2^/g)	3786		
Setting time (min)	Initial setting	204 min	Min 60 min for initial setting
	Final setting	247 min	
Soundness (mm)	1.0		< 10
Compressive strength (N/mm^2^)	Early strength (2 days)	Later strength (28 days)	> 10 for compressive strength 2 days > 42.5 for compressive strength 28 days
	20.6	43	

Chemical components	Percent (wt%)	EN 197-1/2013^22^ Specification limits	
SiO_2_	22.9		
Al_2_O_3_	6.07		
Fe_2_O_3_	6.83		
CaO	54.33		
MgO	3.96		
SO_3_	2.01	< 3.5	
K_2_O	1.1		
Na_2_O	0.31		
Cl	0.04	≤ 0.1%	
Insoluble residue	1.50	≤ 5.0%	
L.O.I	1.40	≤ 5.0%

### Glass powder

**Fig. 2:**
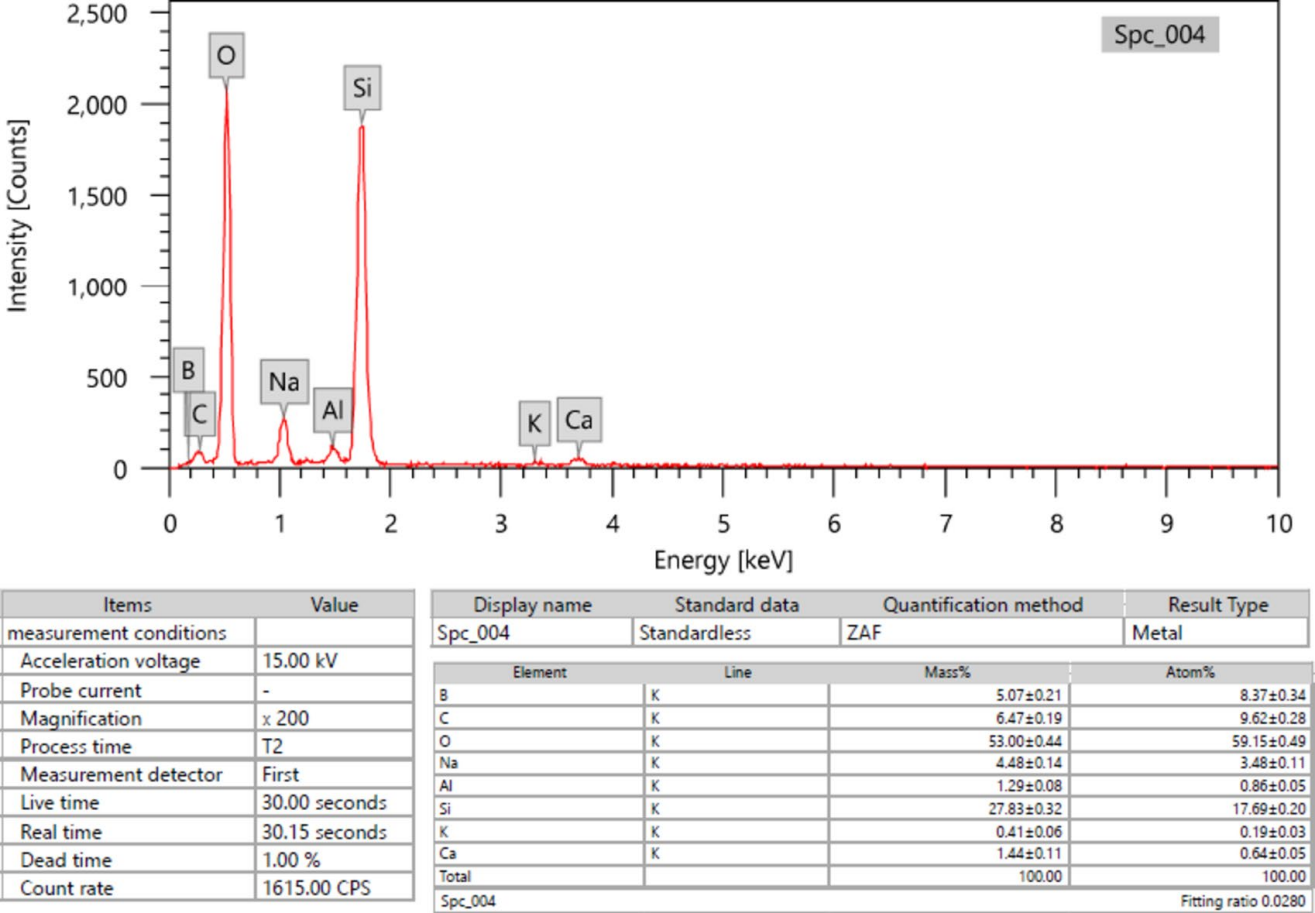
Energy dispersive X-ray analysis (EDX) of glass.

Damaged laboratory glassware was collected from chemistry laboratories, and then it was cleaned and sterilized. At first, waste glass was broken into smaller pieces manually. Grinding was carried out using a ball mill with different-sized balls, high-speed rotation, and high pressure. The milling process continued till the sample reached a fine powder, which makes it suitable for use in mortar. Figure 2 provides a summary of the EDX analysis of WGP.

**Fig. 3:**
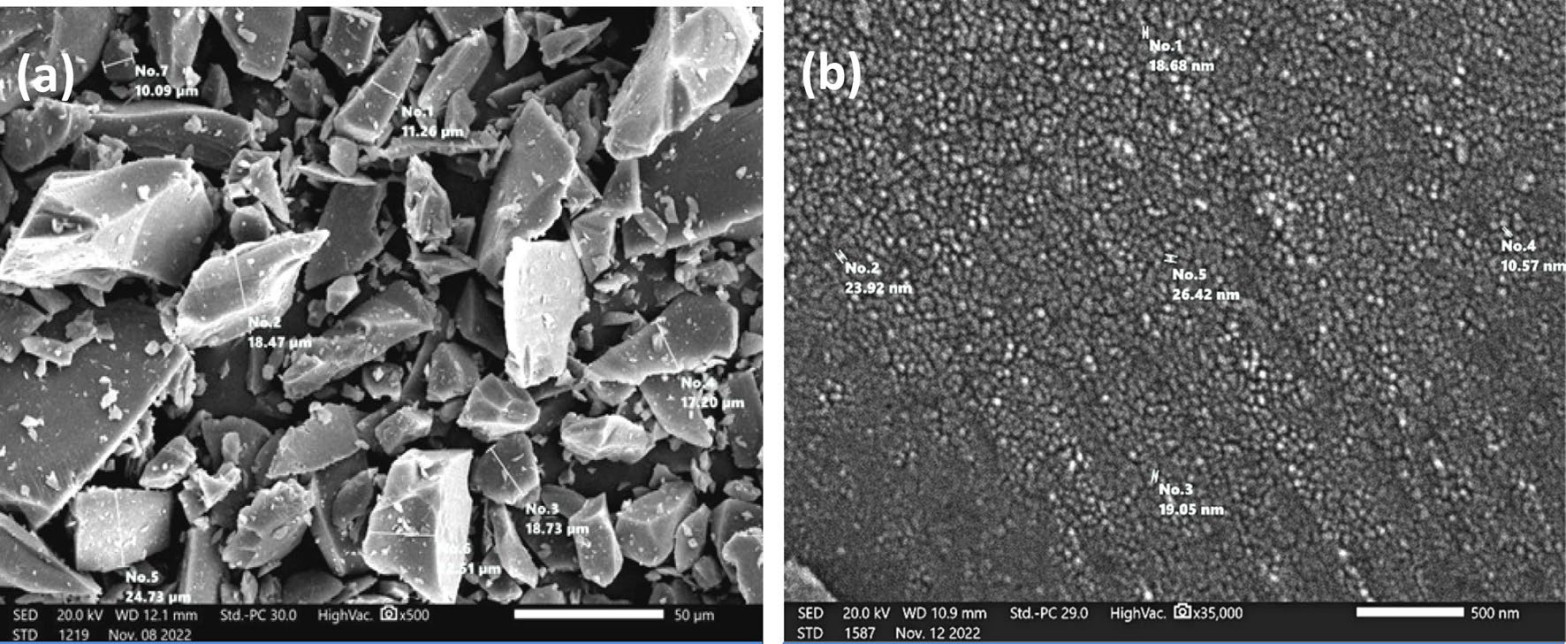
(**a**) Shape and size of the micro-GP particles seen by SEM. (**b**) Shape and size of the nano-GP particles seen by SEM.

Two different sizes of glass particles were utilized in this study. Scanning electron microscope (SEM) analysis confirmed that the micro particles had a size of 10.09–24.73 μm, as shown in Fig. 3a, while the nano particles were found to have a particle size of 10.57–26.42 nm, as shown in Fig. 3b.

### Mixture design

Mortar mixture was designed with a fixed water-cement ratio of 0.485 and a ratio of fine material (sand) to cement of 2.75. This included a plain control mortar designated as M0. Two different sizes of waste glass powder were studied: micro glass particles (particles less than 75 μm) with a size range of 10.09–24.73 μm and nano particles, which have a particle size of 10.57–26.42 nm. Waste glass powder was used as a cement replacement or cement addition by 0%, 5%, 10%, 20%, and 30% by weight. The code name of mixes is based on the size of waste glass and the percentage of replacement or addition. For example, mix M20-NR-WG refers to a mortar mix prepared with 20% of nano waste glass as a partial replacement for cement, and mix M 20-MA-WG refers to a mortar mix prepared with 20% of micro waste addition of cement. The composition of the mixture is summarized in Table 2.

**Table 2:** Proportion of mortar mix design (ratio by weight of cement).

Code	Glass powder%	Cement%	Water/cement ratio	Sand/cement ratio
M0	0	100	0.485	2.75
Micro glass (M)/Nano glass (N)				
M5-MR/NR-WG	5%	95	0.485	2.75
M10-MR/NR-WG	10%	90	0.485	2.75
M20-MR/NR-WG	20%	80	0.485	2.75
M30-MR/NR-WG	30%	70	0.485	2.75
M5- MA/NA-WG	5%	100	0.485	2.75
M10-MA/NA-WG	10%	100	0.485	2.75
M20 -MA/NA-WG	20%	100	0.485	2.75
M30-MA/NA-WG	30%	100	0.485	2.75

### Sample preparation

The samples were mixed using the following procedure: sand was put in the mixer, then cement and waste glass were added and dry mixed for 30 s to ensure a homogeneous distribution. Water was added, and mixing was conducted as follows; fast mixing for 1 min, pause for 1 min, and finally, fast mixing for 1 min. Cubic specimens of 50 × 50 × 50 mm were cast for compressive strength test, and prisms of 40 × 40 × 160 mm were cast for flexural strength tests. A disc of 50 mm in diameter was cast for radiation shielding evaluations. All specimens were cured in a water bath until the day of testing.

## Methodology

### Mechanical properties

The density of hardened mortar was calculated according to BS 1881: Part 114@@^39^. The mechanical properties of mortar mixes were evaluated in terms of the compressive strength test of 50 × 50 × 50 mm cube samples after 7 and 28 days of curing and the flexure strength of 40 × 40 × 160 prism samples at a curing age of 28 days, according to ASTM C109 and ASTM C348-21@^40,41^, respectively. A three-point bending test was performed to determine the flexure strength of mortar. The distance between the points of support was 120 mm. The compressive strength of the mortar was determined from the specimen halves obtained from bending tests. Note that the load was applied at a uniform rate of 15 N/s, according to EN 1015-11@@^42^. All the experiments were carried out on three specimens, and the mean values were presented in the results.

### Attenuation measurements

**Fig. 4:**
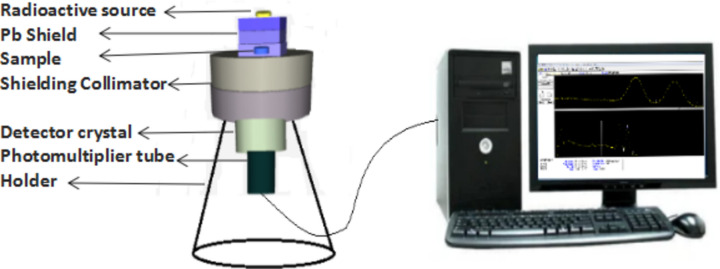
Configuration of the experimental setup.

To assess the shielding effectiveness and penetration of gamma rays through a material, a NaI detector utilizing scintillation principles was employed. Radioactive sources emitting energies ranging from 0.0595 to 1.332 MeV^43–45^ were utilized for this analysis; their specifications are shown in Table 3.The experimental device's configuration is shown in Fig. 4. This experiment involved the emission of rays from a gamma source, which penetrated the glass-mortar sample before being received by the detector. When gamma rays interact with the NaI crystal, they produce scintillation light. Photomultiplier tubes (PMTs) detect this light and convert it into signals. WIN-TMC software was used to process these pulses and construct energy spectra, which represent the distribution of gamma-ray energies detected by the NaI detector. Data analysis shows that the dead time ranges from 1 to 5% in all samples. To enhance the sensitivity and accuracy of the analyzed data, the spectrums were analyzed again using Genie 2000 software to check the area under the peaks, the real time, and the count rate.

**Table 3:** The sources used and their specifications.

Radioactive source	Energy (MeV)	Half-life time (day)
Am-241	0.05951	157.850
Ba-133	0.08099	3847.91
	0.354	
Eu-152	0.121	4943.29
Cs-137	0.661	11004.98
Co-60	1.173	1925.31
	1.332

### Shielding parameters

The linear attenuation coefficient (LAC) is defined as the interaction probability between gamma photons and shielding material per unit path length and computed according to Beer–Lambert’s law^46^ as follows:

1 $${\text{LAC}} = {\upmu } = \frac{ - 1}{{\text{ x}}}\ln \left( {\frac{{\text{ I}}}{{{\text{I}}_{o} }}} \right)$$

where µ is the linear attenuation coefficient (cm^−1^), ${I}_{o}$ is intensity of the incident beam, I refers to transmitted beam intensity and x refers to thickness of the shielding material.

The ratio of the linear attenuation coefficient over composite density (*ρ*) is 0known as the mass attenuatio n coefficient (MAC).

2 $${\text{MAC}} = \mu_{m} = \frac{\mu }{\rho }$$

where ${\mu }_{m}$ is the mass attenuation coefficient (${\text{cm}}^{2}/\text{g}$).

The relative deviations for the measured mass attenuation coefficient compared to the XCOM result (Δ_1_%) and between micro and nano linear attenuation coefficient results (Δ_2_%) are given by the following Eqs. (*47*):

3 $${\Delta }_{1} \% = \frac{{{\text{MAC}}_{{{\text{Exp}}}} { } - {\text{MAC}}_{{{\text{XCOM}}}} }}{{{\text{MAC}}_{{{\text{XCOM}}}} }} \times 100$$

4 $${\Delta }_{2} \% = \frac{{{\text{LAC}}_{{{\text{Nano}}}} { } - {\text{LAC}}_{{{\text{Micro}}}} }}{{{\text{LAC}}_{{{\text{Micro}}}} }} \times 100$$

Equation (5) describes the concept of the half-value layer (HVL)^48^, which represents the thickness required to reduce the intensity of incoming photons by half. Similarly, the tenth value layer (TVL) refers to the thickness needed to reduce the incident beam's intensity to 90% of its original value. The calculation for the TVL can be derived by Eq. (6)^@^^49^.

5 $$\text{HVL}=\frac{\text{ln}(2)}{\upmu }$$

6 $${\text{{\rm T}VL}} = \frac{{\ln \left( {10} \right)}}{{\upmu }}$$

The mean free path (MFP), which is described by Eq. (7)@^50^, is the average distance between two photon collisions^51^. To clarify the chemical composition of a photon shield and examine the relative changes in photon absorption processes with energy for various shields, the effective atomic numbers (Z_eff_) are used as shown in Eq. (8)@^52^.

7 $$MFP=\frac{1}{\upmu }$$

8 $${\rm Z}_{eff}=\frac{{\sum }_{i}{w}_{i}{A}_{i}{(\frac{\mu }{\rho })}_{i}}{{\sum }_{i}{w}_{i}\frac{{A}_{i}}{{z}_{i}}{(\frac{\mu }{\rho })}_{i}}$$

where w_i_, A_i_, and Z_i_ are refers to weight fraction, atomic weight, and the atomic number of each constituent element in each specimen, respectively.

## Results and discussion

### Mechanical properties

Table 4 shows the influence of waste glass powder on mortar density. It is observed that as the percentage of waste glass powder increases, so does the measured density of the composite. This due to the ultrafine-size waste glass powder particles fill the voids between the cement particles, leading to a higher packing density. In general, a higher packing density means particles are closer together, creating a stronger composite, which is desirable for enhancing efficiency and durability.

**Table 4:** Density values for the investigated samples.

Glass percent	Density (g cm^−3^)			
0%G	1.65			

	Micro		Nano	
	Replacement	Additive	Replacement	Additive
5%G	1.69	1.72	1.71	1.74
10%G	1.73	1.77	1.76	1.80
20%G	1.81	1.84	1.83	1.89
30%G	1.89	1.91	1.90	1.94

**Fig. 5:**
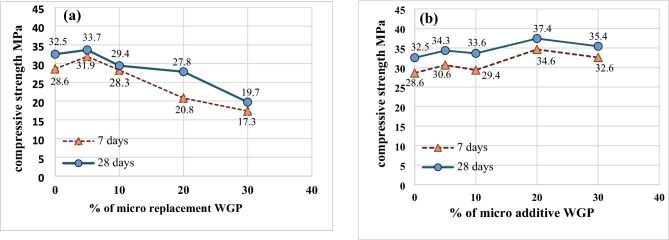
(**a**) Effect of micro replacement WG on compressive strength. (**b**) Effect of micro additive WG on compressive strength.

The compressive strength of different mortar specimens containing micro waste glass powder at 7 and 28 days is shown in Fig. 5. In Fig. 5a, it was observed that there is a decrease in compressive strength by incorporating micro waste glass powder as cement replacement; this reduction was 9.4, 14.3, and 39.3% at 28 days for 10, 20, and 30% replacement, respectively. slightly improved compressive strength by 3.5% for M5-MR-WG; these results conform with previous research^53^. The reduction in compressive strength for a high percentage of WGP replacement may be due to the smooth surface of glass particles^54^. On the other hand, Fig. 5b shows improvement in 7- and 28-day compressive strength by increasing the percentage of micro WGP as a cement addition. The increase in 28-day compressive strength was 5.6, 3.4, 15, 9.0 for 5, 10, 20, and 30% addition of WGP, respectively. The highest improvement of 28 days in compressive strength was achieved with a 20% addition of micro WGP; this finding conforms with^55^. This improvement may be due to the pozzolanic reaction of micro glass powder, which produces C–S–H gel^56^.

**Fig. 6:**
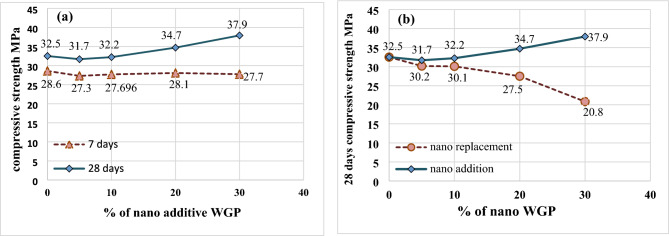
(**a**) Effect of nano additive WG on compressive strength. (**b**) Difference between effect of both methods of using nano WG on compressive strength.

The effect of nano WGP on 7 and 28-day compressive strength is shown in Fig. 6 for mortar mixes with nano WGP as an addition. As expected, the compressive strength increased with time and with the addition of nano. This is because of the reactivity of nano particles to form a C–S–H gel as a result of the pozzolanic reaction and its filler effect^57,58^. The addition of nano WGP led to an increase in the 28-day compressive strength by 7 and 17% for 20 and 30% of WGP, respectively. On the other hand, the replacement of cement with nano WGP decreased the compressive strength by 7.4, 15.4, and 39.8% for 10%, 20%, and 30%, respectively, as shown in Fig. 6b. This reduction was almost the same as when using micro WGP replacement. The possible reason was that the replacement of a high percent of cement with WGP led to an acceleration in cement hydration and a consequently insufficient amount of released alkalis, which did not make it possible to compensate for the incomplete hydration of cement^59,60^. Another explanation for this reduction was found in recent research, which revealed that the high dose (higher than 12.5%) of silica glass powder leads to a decrease in mechanical strength because the chemical bonds of the C–S–H gel can be minimized with a lower amount of cement (replaced by silica glass powder) in the mortar^61^.

**Fig. 7:**
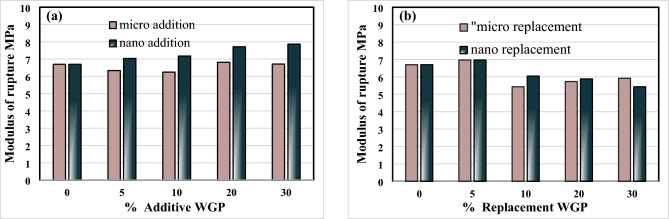
(**a**) Flexure strength for additive WGP. (**b**) Flexure strength for replacement WGP.

Figure 7a shows the flexure strength of mortar specimens incorporating micro or nano WGP addition. There is a slight improvement in the flexure strength of mortar containing 5% and 10% nano additive WGP by 5, and 7%, respectively, while increasing the percentage of addition to 20% and 30%, the flexure strength increased by 15, and 16% compared to the flexure strength of the control mix. In general, the addition of micro/nano WGP has a filling effect that enhances bond strength and hardness, resulting in improved resistance to applied loads and increased flexural strength^62,63^. Figure 7b shows the flexure strength of mortars containing micro or nano replacement WGP. The highest flexure strength was observed in specimens with 5% replacement WGP, while the flexural strength decreased with replacement cement with WGP higher than 5%. This reduction in flexural strength was due to the excess of WGP as an inert material in the matrix, and a secondary cementitious gel was not formed^64^.

### Radiation shielding evaluation

Table 5 presents the variations in MAC, theoretical XCOM, and relative deviation values of micro glass-mortar samples obtained from both the replacement and additive methods. The MAC values for all samples can be observed to decrease with increasing photon energy. The reason for this is that the energy levels are divided into three regions: low energies, medium energies, and high energies. At low photon energies, the most common interaction is the photoelectric effect, which is proportional to $\frac{{\text{z}}^{\text{n}}}{{\text{E}}^{3.5}}$, n = 4–5, while the chance of interaction with Compton scattering is proportional to ${\text{E}}^{-1}$, even though it is the predominant interaction at intermediate energies. Above 1.022 MeV pair production is the dominant interaction, where the chance of contact is proportional to ${\text{E}}^{2}$. The results showed that the glass-mortar samples prepared by the addition method had a greater absorption of photons than their analogues prepared by the replacement method. To verify the validity of the results, Table 5 compares the theoretical values of XCOM with the experimental mass attenuation coefficient (MAC) values obtained using the two methods (replacement and addition). Good compatibility has been shown between the theoretical and experimental data, where the relative deviation (Δ_1_%) results don’t exceed 2.88%.

**Table 5:** Mass attenuation coefficients, theoretical XCOM, and relative deviation values for free mortar, replacement, and additive micro glass-mortar mixtures in different concentrations.

Glass percent	Energy (keV)	Micro					
		MAC, ${\text{cm}}^{2}/\text{g}$		${\Delta }_{1}$%	MAC, ${\text{cm}}^{2}/\text{g}$		${\Delta }_{1}$%
		(Exp)	(XCOM)		(Exp)	(XCOM)	
0%	59.53	0.26616	0.26520	0.36			
	80.99	0.18959	0.19100	− 0.74			
	121.78	0.15023	0.15020	0.02			
	354	0.10165	0.09989	1.76			
	661	0.07841	0.07702	1.81			
	1173	0.05879	0.05867	0.20			
	1332	0.05447	0.05500	− 0.96			
	**Replacement**				**Additive**		
5%	59.53	0.26200	0.26270	− 0.26	0.26261	0.26500	− 0.90
	80.99	0.18639	0.19010	− 1.95	0.18868	0.19100	− 1.22
	121.78	0.14979	0.14990	− 0.07	0.14732	0.15020	− 1.92
	354	0.10068	0.09988	0.80	0.09875	0.09988	− 1.14
	661	0.07704	0.07702	0.03	0.07683	0.07702	− 0.25
	1173	0.05835	0.05867	− 0.54	0.05874	0.05867	0.13
	1332	0.05428	0.05500	− 1.31	0.05399	0.05500	− 1.84
10%	59.53	0.26169	0.26070	0.38	0.26195	0.26480	− 1.07
	80.99	0.18524	0.18930	− 2.14	0.18728	0.19090	− 1.90
	121.78	0.14855	0.14970	− 0.77	0.14706	0.15010	− 2.03
	354	0.09881	0.09987	− 1.06	0.09861	0.09987	− 1.27
	661	0.07697	0.07701	− 0.06	0.07668	0.07701	− 0.43
	1173	0.05768	0.05867	− 1.70	0.05844	0.05866	− 0.38
	1332	0.05395	0.05500	− 1.90	0.05405	0.05499	− 1.71
20%	59.53	0.25474	0.25650	− 0.69	0.26078	0.26450	− 1.41
	80.99	0.18629	0.18770	− 0.75	0.18701	0.19080	− 1.99
	121.78	0.14824	0.14930	− 0.71	0.14672	0.15010	− 2.25
	354	0.09869	0.09985	− 1.16	0.09859	0.09986	− 1.27
	661	0.07646	0.07701	− 0.71	0.07633	0.07700	− 0.87
	1173	0.05759	0.05866	− 1.82	0.05813	0.05865	− 0.88
	1332	0.05377	0.05499	− 2.22	0.05386	0.05499	− 2.06
30%	59.53	0.25388	0.25240	0.59	0.26073	0.26410	− 1.27
	80.99	0.18484	0.18620	− 0.73	0.18665	0.19060	− 2.07
	121.78	0.14795	0.14880	− 0.57	0.14642	0.15000	− 2.38
	354	0.09848	0.09983	− 1.35	0.09843	0.09984	− 1.41
	661	0.07637	0.07700	− 0.81	0.07606	0.07699	− 1.21
	1173	0.05736	0.05866	− 2.22	0.05756	0.05864	− 1.84
	1332	0.05375	0.05499	− 2.26	0.05340	0.05498	− 2.88

The concept of effective atomic number in composite materials made up of multiple elements,is not an integer like atomic number but rather a numerical quantity. It is calculated based on or percentages of each element present in the composite material^65^.

**Fig. 8:**
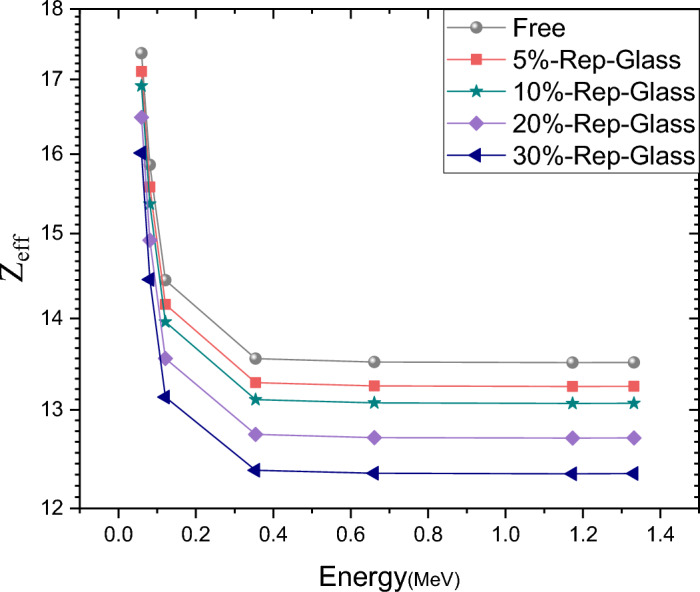
The relationship between photon energy and the variations in effective atomic number (${Z}_{eff}$) values.

Figure 8 shows the effective atomic number as a function of energy. It is observed that there is a decrease in effective atomic number (Z_eff_) with increasing energy levels. This depends on the mass attenuation coefficient (MAC) and the difference in the way gamma photons interact with matter at this level. Whether by photoelectric effect, Compton scattering, or pair production, It is also noted that an increase in the glass concentration in the composite material leads to a decrease in Z_eff_. This observation can be attributed to glass having elements with a lower atomic number compared to other elements found in mortar. So as the proportion of glass increases, it dilutes the overall average atomic number of the composite, hence reducing Z_eff_.

**Fig. 9:**
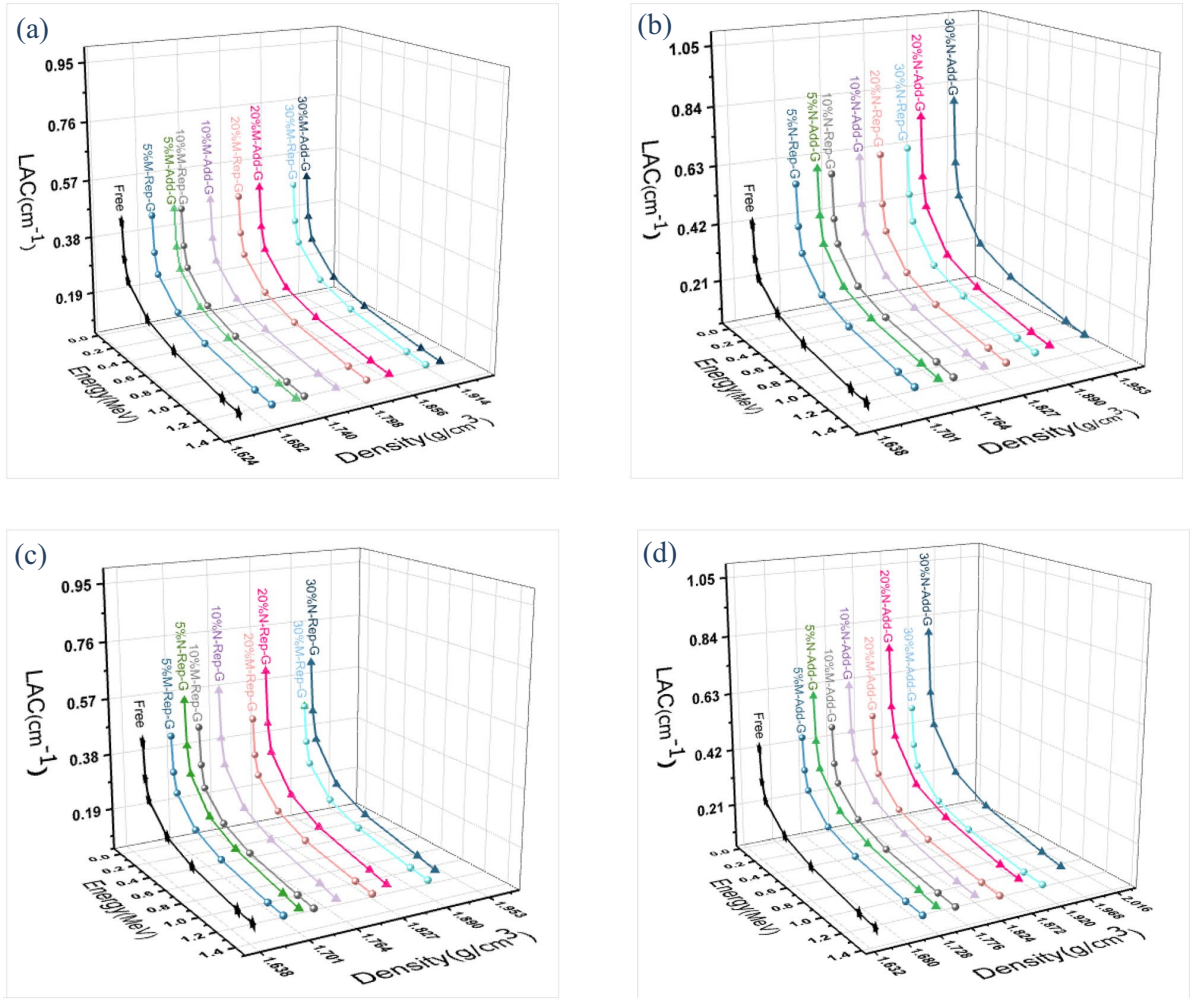
(**a**) The variation of LAC values of mortar-micro glass as a function of energy and density by both methods. (**b**) The variation of LAC values of mortar-nano glass as a function of energy and density by both methods. (**c**) The variation of LAC values of mortar-micro/nano glass as a function of energy and density by replacement method. (**d**) The variation of LAC values of mortar-micro/nano glass as a function of energy and density by addition method.

**Fig. 10:**
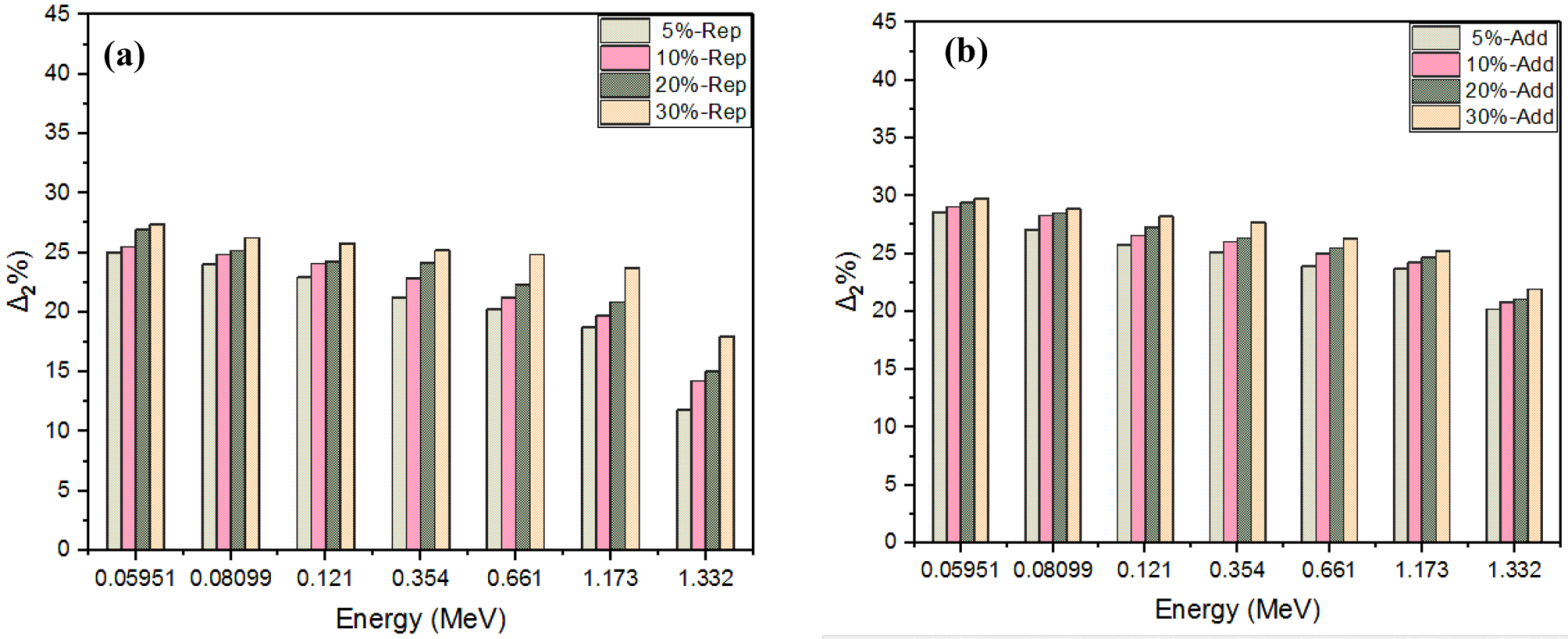
(**a**) Relative deviation Δ% between the micro and nano values for the LAC by replacement method. (**b**) Relative deviation Δ% between the micro and nano values for the LAC by addition method.

Figure 9a–d present a plot of the linear attenuation coefficient (LAC) against increasing energy and density. The LAC values for all tested compounds have been observed to decrease with rising energy. This is because of the higher penetrating capability of high-energy photons. Since the linear attenuation coefficient is a density-dependent parameter, its value increases with increasing glass content in the composite. Also, Figs. 9c and 9d show that in both methods of replacement and addition, nano-sized composites have higher LAC results compared to micro-sized composites. Besides, the addition method gave the best results of all. These observations are all related to the increasing density. For example, at an energy level of 0.0595 MeV, the LAC of the M10-MR-WG increases from 0.459 to 0.473 cm^−1^ in M10-MA-WG, and the density increases from 1.73 to 1.77 g/${\text{cm}}^{3}$, respectively, while it increases to 0.623 cm^−1^ in nano-additive glass with a density of 1.8 g/cm^3^. Figure 10 elucidate the relative increasing rate Δ_2_% in the linear attenuation coefficients between the micro and nano particles as a function of energy (MeV) at varied glass percentages.

The results show that the relative increase rate Δ_2_% between the micro and nano in LAC values increases with increasing glass concentration and declines as the photon energy increases. For example, the deviation in M5-NA-WG was 10.39% at 0.05951 MeV, compared to 11.34% in M30-NA-WG at the same energy. The deviation for the maximum examined energy (1.332 MeV) was 3.32% for M5-NA-WG, compared to 3.1% for M30%-NA-WG. To fully assess shielding efficiency against gamma-ray photons, Tables 6 and 7 show the results of HVL, MFP, and TVL by both methods of replacement and addition, respectively, at various energy levels up to 1332 keV and glass concentrations ranging from 0 to 30%. It turns out that for all the examined samples, HVL, TVL, and MFP increase with increasing energy. For instance, at a concentration of 20% micro-replacement glass, the HVL values were 1.50, 2.06, 2.56, 3.79, 5.01, 6.50, and 6.99 cm at energy levels of 59.53, 80.99, 121.78, 354, 661, 1173, and 1332 keV, respectively. Also, we observed that the results decreased with our transition from 0% glass to 30% glass, depending on the density rising, either for micro or nano particles, where the sample with 0% glass content gave the highest values. For example, at an energy level of 59.53 keV, the MFP values of micro-replacement-glass samples were 2.28, 2.26, 2.18, 2.17, and 2.08 cm for concentrations of 0%, 5%, 10%, 20%, and 30%, respectively. While they were 2.28, 2.21, 2.03, 2.21 and 1.99 cm for micro-additive glass samples. Further, it is observed that samples prepared with the addition method have a more positive effect on attenuation than their analogs prepared with the replacement method. On the other hand, the results showed that the particles that were ground into nano-sized particles achieved better performance in shielding than the micro-sized particles. That is because glass nano particles narrow the voids between molecules of the nano composite and hence increase the collisions between the photons and the nano particles, causing more attenuation. For example, at an energy level of 1332 keV, the TVL of M30-NA-WG was 14.82 cm, while it was 22.30 cm for M30-MA-WG.By comparing all the results of the tested samples, it was obvious that M30-NA-WG exhibited the lowest values of HVL, TVL, and MFP, and it is known that smaller values of HVL, TVL, and MFP mean a better shield.

**Table 6:** HVL, MFP, and TVL values for micro and nano glass-mortar mixture at different energy levels by replacement method.

Energy (keV)		Replacement method								
		0%G	5%G		10%G		20%G		30%G	
			Micro	Nano	Micro	Nano	Micro	Nano	Micro	Nano
HVL(cm)	59.53	1.58	1.57	1.24	1.51	1.20	1.50	1.11	1.44	1.09
	80.99	2.22	2.16	1.71	2.09	1.67	2.06	1.57	1.95	1.50
	121.78	2.80	2.71	2.21	2.70	2.12	2.56	2.00	2.45	1.89
	354	4.13	4.07	3.36	3.97	3.23	3.79	2.98	3.67	2.87
	661	5.36	5.32	4.42	5.21	4.22	5.01	3.94	4.77	3.70
	1173	7.15	6.97	5.88	6.80	5.60	6.50	5.22	6.27	4.76
	1332	7.71	7.41	6.56	7.30	6.33	6.99	5.80	6.71	5.27
MFP(cm)	59.53	2.28	2.26	1.79	2.18	1.73	2.17	1.61	2.08	1.58
	80.99	3.20	3.11	2.46	3.01	2.41	2.97	2.26	2.82	2.16
	121.78	4.03	3.92	3.19	3.89	3.05	3.70	2.89	3.54	2.73
	354	5.96	5.88	4.85	5.73	4.66	5.46	4.30	5.29	4.15
	661	7.73	7.68	6.37	7.51	6.08	7.23	5.68	6.88	5.33
	1173	10.31	10.06	8.48	9.81	8.08	9.37	7.53	9.04	6.86
	1332	11.13	10.68	9.46	10.53	9.14	10.08	8.37	9.69	7.60
TVL(cm)	59.53	5.24	5.20	4.13	5.01	3.99	4.99	3.70	4.80	3.64
	80.99	7.36	7.17	5.67	6.94	5.56	6.83	5.21	6.48	4.97
	121.78	9.29	9.02	7.33	8.96	7.03	8.51	6.66	8.15	6.28
	354	13.73	13.53	11.16	13.19	10.74	12.58	9.91	12.18	9.54
	661	17.80	17.69	14.67	17.29	14.01	16.64	13.09	15.84	12.28
	1173	23.74	23.16	19.52	22.59	18.61	21.58	17.33	20.82	15.80
	1332	25.62	24.60	21.79	24.24	21.04	23.22	19.27	22.30	17.50

**Table 7:** HVL, MFP, and TVL values for micro and nano glass-mortar mixture at different energy levels by the addition method.

Energy (keV)		Addition method								
		0%G	5%G		10%G		20%G		30%G	
			Micro	Nano	Micro	Nano	Micro	Nano	Micro	Nano
HVL(cm)	59.53	1.58	1.53	1.15	1.46	1.11	1.40	0.93	1.38	0.88
	80.99	2.22	2.10	1.60	2.06	1.53	1.95	1.32	1.93	1.24
	121.78	2.80	2.66	2.06	2.58	1.97	2.50	1.67	2.45	1.59
	354	4.13	4.07	3.13	3.90	2.98	3.74	2.55	3.66	2.37
	661	5.36	5.24	4.07	5.11	3.90	4.94	3.30	4.73	3.09
	1173	7.15	6.86	5.35	6.62	5.33	6.48	4.44	6.30	4.11
	1332	7.71	7.27	6.00	7.08	5.64	6.88	4.78	6.61	4.46
MFP(cm)	59.53	2.28	2.21	1.66	2.11	1.61	2.03	1.34	1.99	1.27
	80.99	3.20	3.03	2.30	2.97	2.21	2.82	1.91	2.78	1.79
	121.78	4.03	3.84	2.98	3.73	2.84	3.60	2.40	3.54	2.29
	354	5.96	5.88	4.52	5.62	4.30	5.39	3.68	5.28	3.43
	661	7.73	7.55	5.88	7.37	5.62	7.12	4.76	6.82	4.46
	1173	10.31	9.90	7.72	9.55	7.69	9.35	6.41	9.10	5.93
	1332	11.13	10.49	8.65	10.21	8.14	9.93	6.89	9.54	6.43
TVL(cm)	59.53	5.24	5.10	3.82	4.87	3.70	4.66	3.09	4.59	2.92
	80.99	7.36	6.97	5.30	6.84	5.09	6.48	4.39	6.40	4.13
	121.78	9.29	8.85	6.85	8.58	6.55	8.30	5.53	8.14	5.27
	354	13.73	13.53	10.41	12.95	9.91	12.42	8.48	12.17	7.89
	661	17.80	17.39	13.53	16.97	12.95	16.40	10.95	15.70	10.28
	1173	23.74	22.79	17.78	21.99	17.70	21.53	14.76	20.94	13.66
	1332	25.62	24.15	19.92	23.51	18.74	22.85	15.86	21.97	14.82

Table 8 presents a comparison of half-value layers (HVL) for lead-mortar and glass-mortar using both replacement and additive methods at various energy levels and concentrations. Table 8 emphasizes the effectiveness of glass in mortar as an environmentally friendly shielding material. The value of the MAC of the lead-mortar mixture was calculated using XCOM software to evaluate HVL theoretically and compare it with the HVL of the glass-mortar mixture. And it was found, for instance, that for radiation with an energy of 0.08099 MeV, 10% wt. lead in the mixture and a half-thickness of 1.51 cm are required. And for the same energy level, 10% wt. of additive glass in the mixture with a half-thickness of 2.10 cm is needed. It is therefore preferable to use a higher thickness of an eco-friendly material rather than a lower thickness of toxic lead.

**Table 8:** HVL for lead-mortar, replacement, and additive glass-mortar at different energy levels and different concentrations.

Energy (keV)	$$X_{{{\raise0.7ex\hbox{$1$} \!\mathord{\left/ {\vphantom {1 2}}\right.\kern-0pt} \!\lower0.7ex\hbox{$2$}}(_{Lead} )}}$$				$$X_{{{\raise0.7ex\hbox{$1$} \!\mathord{\left/ {\vphantom {1 2}}\right.\kern-0pt} \!\lower0.7ex\hbox{$2$}}(_{Replacement glass} )}}$$				$$X_{{{\raise0.7ex\hbox{$1$} \!\mathord{\left/ {\vphantom {1 2}}\right.\kern-0pt} \!\lower0.7ex\hbox{$2$}}(_{Additive glass} )}}$$			
	5%	10%	20%	30%	5%	10%	20%	30%	5%	10%	20%	30%
59.51	1.13	0.96	0.73	0.58	1.57	1.51	1.50	1.44	1.53	1.46	1.40	1.38
80.99	1.7	1.51	1.25	1.05	2.16	2.09	2.06	1.95	2.10	2.06	1.95	1.93
121	1.9	1.55	1.13	0.89	2.71	2.70	2.56	2.45	2.66	2.58	2.50	2.45
354	3.57	3.47	3.29	3.12	4.07	3.97	3.79	3.67	4.07	3.90	3.74	3.66
661	4.71	4.66	4.57	4.47	5.32	5.21	5.01	4.77	5.24	5.11	4.94	4.73
1173	6.21	6.18	6.1	6.03	6.97	6.80	6.50	6.27	6.86	6.62	6.48	6.30
1332	6.63	6.59	6.52	6.44	7.41	7.30	6.99	6.71	7.27	7.08	6.88	6.61

## Conclusion

A comprehensive study was conducted to investigate the impact of particle size in waste glass powder (WGP) on the mechanical and shielding properties of cement-based materials. The study included an analysis of physical and mechanical properties, as well as attenuation tests. The major findings are illustrated below:

1. The density of the mortar increased with the addition of a higher percentage of waste glass powder, regardless of the lower specific gravity of the glass. However, the ultrafine size of nano WGP has a packing effect and consequently improves the density.
2. The mechanical properties improved significantly. At the 5% replacement ratio, the compressive strength increased by up to 3.5%, and the flexural strength increased by 5%. By increasing the percentage of micro or nano WGP up to 5%, the compressive strength and flexure strength decreased significantly.
3. The opportunities for enhancing the overall mechanical properties were to use WGP as an additive material, where the improvement in 28-day compressive strength was 5.6, 3.4, 15, 9.0 for 5, 10, 20, and 30% additive WGP, respectively. According to the results, the highest improvement of 28 days in compressive strength was achieved with a 20% addition of micro WGP.
4. Particle size plays an important role in using WGP as an additive in cement-based materials, whereby increasing the ratio of the percentage of additive nano WGP to 30%, the compressive strength increased up to 17%. This is due to the high reactivity of nano-silica as a pozzolanic and filler material.
5. Flexural strength for mortars containing WGP as a cement replacement is lower than that of control mortars, except for 5% of WGP replacement. Also, as with the same finding in compressive strength, using 20 or 30% nano additive WGP cement enhanced the flexure strength up to 15 and 17%, respectively.
6. The mass attenuation coefficient values between experimental data and theoretical XCOM data were in great agreement.
7. The mortar sample with 30% nano additive WGP had the highest LAC value. Following it are samples with 30% micro-additive WGP, 30% nano-replacement WGP, and 30% micro replacement WGP, in that order. The lowest value of LAC in these samples was an ordinary mortar sample.
8. The data show a drop in HVL, TVL, and MFP as we move from 0 to 30% glass, whether for micro or nano particles, due to the increase in density. Overall, the mortar sample with 30% nano additive WGP demonstrated the greatest attenuation performance among the evaluated samples.

## Data Availability

All data generated or analysed during this study are included in this published article.
